# Feeling connected to nature: validation of the connectedness to nature scale in the Italian context

**DOI:** 10.3389/fpsyg.2023.1242699

**Published:** 2023-10-12

**Authors:** Chiara Lovati, Federico Manzi, Cinzia Di Dio, Davide Massaro, Gabriella Gilli, Antonella Marchetti

**Affiliations:** ^1^Department of Psychology, Research Unit on Psychology of Art, Università Cattolica del Sacro Cuore, Milan, Italy; ^2^Department of Psychology, Research Unit on Theory of Mind, Università Cattolica del Sacro Cuore, Milan, Italy; ^3^Department of Psychology, Research Unit on Robopsychology in the Lifespan, Università Cattolica del Sacro Cuore, Milan, Italy

**Keywords:** connectedness to nature scale, CNS, Italian validation, environment, proenvironmental behaviors

## Abstract

Environmental issues are at the center of the political and cultural debate, representing one of the greatest challenges of our century. Sustainability and pro-environmental conducts are recognized as increasingly urgent to address the decay of ecosystems. To support the acquisition of attitudes that give greater consideration to environmental issues, experiencing a sense of connection with nature has been acknowledged in psychology as a particularly relevant individual component. Among the most commonly used scales in Anglo-Saxon context to analyses this feeling is the Connectedness to Nature Scale (CNS) assessing the emotional and experiential bond between humans and nature. To examine the reliability and validity of this scale in the Italian context, a study including 271 Italian adults (44,3% female; 55% males; Mean age = 34.70; SD = 13.584; age-range = 18-65 years) was conducted to establish evidence supporting the internal consistency of the CNS, as well as its ability to measure convergent, discriminant, and predictive validity. A Confirmatory Factor Analysis showed that CNS in Italian has a single-factor structure as reported in the original version by Mayer and Frantz. Furthermore, as expected, positive correlations were observed between the CNS and pro-environmental attitudes and negative correlations with civic moral disengagement. Finally, as assumed, the CNS positively correlated with mental well-being. A broad vision of this study concerns the idea that individuals who have a stronger connection with nature are likely to exhibit reduced tendencies to cause harm to it.

## 1. Introduction

At present, environmental issues pose one of the foremost challenges for contemporary human civilization (Barrera-Hernández et al., 2020). On a daily basis, we witness both first-hand and through media reports around the world the effects of climate change on ecosystems and the dramatic social and economic consequences. This is leading, although at different times and to different extents, to the promotion at national and international levels of policies trying to contain these effects and support new perspectives on experiencing the natural environment. A growing number of disciplines, including psychology, are actively exploring the interplay between humans and nature and investigating how this connection can impact eco-sustainable behaviors (Nisbet and Zelenski, 2013). According to Nisbet et al. (2020) a primarily anthropocentric mindset has led to a detachment between humans and the natural world, thereby contributing to the perpetration of selfish behavior toward the environment and consequently causing an acceleration of the collapse of the current ecosystem balances. Several studies reported high levels of environmental concern in various cultures (Panu, 2020; Ray, 2020), showing that a significant portion of the global population is aware of the dramatic effects of the environmental problems they will face in the present and future, as well as the damage that various human behaviors have caused and are still negatively affecting the environment (Berenguer, 2007). In this context, psychology represents a theoretical and research corpus that contributes substantially to the analysis of the effects of major climate change challenges at both the intra-individual and inter-individual levels; at the same time, it allows for the identification of the most effective strategies to modify behavior and promote a more environmentally responsible lifestyle. Environmental psychology theories are the basis of this study. Numerous research studies have demonstrated that the physical attributes of a natural environment have a direct impact on the psychophysiological well-being or discomfort experienced by individuals (Stern, 2000; Dutcher et al., 2007; Frantz and Mayer, 2014; Arendt and Matthes, 2016). To establish a sort of collaboration and mutual closeness with nature and other forms of life is a natural human propensity, with positive consequences for well-being on the one hand and for the care of the environment on the other. In this context, considering the various psychosocial aspects associated with the connection between humans and nature, Wilson (Gosling and Williams, 2010) introduced the concept of “Biophilia.” Biophilia is described as the “an innate love for the natural world, which humanity should feel universally” (p. 134). In other words, it signifies humans’ inclination to establish a symbiotic relationship with ecosystems, facilitating the development of emotional bonds with nature (Stern, 2000). Based on this premise, the research results suggest that reconnecting humans to natural environments could be an important strategy to address the environmental crisis (Stern, 2000; Arendt and Matthes, 2016). The first research on this topic dates back to the early 1970s. Research on pro-environmental behavior primarily focused on sociodemographic factors, including gender, age, education, marital status, place of residence, and personal economic situation (Capaldi et al., 2014). More recent studies (Berman et al., 2008; Davis et al., 2009; Beery and Wolf-Watz, 2014; Whitburn et al., 2020; Kesenheimer and Greitemeyer, 2021; Seddon et al., 2021) have identified that demographic differences are not always effective in predicting pro-environmental behavior. Consequently, there has been a shift in focus toward psychological factors, encompassing attitudes, beliefs, and subjective norms. The most investigated psychological concepts in relation to environmental behaviors are the attachment to the place (Place attachment), environmental identity (Place identity), and Connection with Nature. Connection to nature, in particular, is conceptualized as a belief about the degree to which people see themselves as part of nature. Hence, fostering a profound sense of connectedness is widely acknowledged as advantageous for environmental preservation and overall sustainability (Tam, 2022). Studies have revealed its predictive nature concerning pro-environmental attitudes, concerns, intentions to act, and comprehensive evaluations of pro-environmental behavior (Zylstra et al., 2014; Tam, 2022). Moreover, it has been posited that individuals with a stronger bond to nature are less inclined to partake in behaviors detrimental to the environment (Mayer and Frantz, 2004). In recent years, various models relating to the connection between humans and the natural world have been introduced in psychology (Mayer and Frantz, 2004; Capaldi et al., 2014; Zylstra et al., 2014; Tam, 2022) resulting in different definitions and theoretical declinations. One of the first was proposed by Mayer and Frantz (2004) that described the connectedness to nature as an individual disposition allowing to evaluate the emotional and experiential connection of an individual to nature. Subsequently, Nisbet and Zelenski (2013) delved deeper into the concept of connection to nature, characterizing it as a relatively enduring personal attribute that remains consistent across time and various situations, encompassing cognitive, affective, and experiential dimensions. Zylstra et al. (2014), on the other hand, defined connection to nature as a stable state of consciousness that involves cognitive, affective, and experiential aspects, fostering a symbiotic relationship between humans and nature. This state is marked by an increased awareness of the interconnectedness between oneself and nature, as well as attitudes and behaviors that align with this state. Despite some updates to the original construct by Mayer and Frantz (2004), the emotional level is central to feeling connected to nature. Additionally, this feeling of connection has been associated with improved mental well-being, including increased positive emotions and decreased mental distress (Capaldi et al., 2014; Panu, 2020).

There is a growing consensus that people in Western countries need to profoundly reshape their behavior and consumption patterns to build an environmentally sustainable society (Mayer and Frantz, 2004; Davis et al., 2009). In this regard, psychology plays a crucial role in understanding the factors that motivate people to develop environmental awareness and subsequently modify their behavior to safeguard the environment (Stern, 2000; Berman et al., 2008; Davis et al., 2009). Various research have shown that connection to nature is an important predictor of involvement in pro-environmental behaviors (Dutcher et al., 2007; Beery and Wolf-Watz, 2014; Whitburn et al., 2020) and fosters more eco-friendly attitudes (Kesenheimer and Greitemeyer, 2021). Moreover, an increased sense of connection to nature is correlated with stronger pro-environmental attitudes, a heightened willingness to participate in sustainable actions, and greater concern regarding the adverse effects of human behavior on the environment (Mayer and Frantz, 2004; Tam, 2013; Nilsson et al., 2019; Nisbet et al., 2020). In Italian context there is also a growing interest in the aforementioned dimensions (Di Fazio and Modica, 2018; Basile and Cavallo, 2020) that requires the development of validated and reliable tools to study the connectedness to nature and their links to psychological well-being and pro-environmental attitudes and behaviors. In response to these challenges, the One Health approach encourages a fundamental shift in perspective, moving away from an anthropocentric view on the environment toward a holistic approach. In recent years, the scientific community has started to examine this approach through a psychological lens, recognizing that psychological well-being is heavily reliant on a healthy natural environment (Gilli et al., 2022). Therefore, it is necessary to explore the consequences of potential climate change with all available tools in order to understand what kind of relationship individuals establish with nature (Picarelli et al., 2016). This needs to be evaluated taking in consideration also the cultural frame reflecting values and attitudes toward the environment. Navarro et al. (2022) for example, conducted a validation study of the CNS scale in seven different countries and, although they found a good level of cross-cultural reliability, they also observed a certain sensitivity of the scale to linguistic and cultural context. Several Italian studies (Picarelli et al., 2016; Berto et al., 2018; Menardo et al., 2020) have used the CNS to carry out research ranging between ecology, environmental education and policies aimed at supporting ecological lifestyles and the well-being of society. Nevertheless, none has ever validated the psychometric properties scale in the Italian context, thus missing to provide evidence in the Italian population for the single-factor model found in the original study (Mayer and Frantz, 2004), as well as of its sensitivity to the Italian language. The purpose of this study is to validate the Connectedness to Nature Scale CNS (Mayer and Frantz, 2004) measuring human’s affective and experiential connection to nature. The study of CNS is based on several foundational assumptions: these include the idea that, to effectively address environmental issues, people need to feel part of the natural world. Specifically, CNS investigates the feeling of being connected and belonging to the larger natural community and the awareness that individual psycho-physical well-being is also related to the general well-being of the natural world. We wonder if CNS (Mayer and Frantz, 2004) once adapted to the Italian language, maintains equivalent psychometric properties to the English version, enabling the measurement of the theoretical construct of connectedness to nature. Other validations of the CNS can be found in literature: Navarro et al. (2022) conducted a first validation in the French context, followed by another one in collaboration with other authors in seven countries (Spain, Netherlands, Turkey, Portugal, Germany, France and Hungary). The latter used a short version of CNS, that showed a good level of reliability and can be considered usable and suitable for cross-cultural research in the domain of human-nature relationship topics (Navarro et al., 2022). To date, there is only a preliminary contribution to the validation of this scale in Italian (Di Fabio and Bucci, 2016). Specifically, the age of the participants ranged from 18 to 20 years (*M* = 18.61, SD 142 = 0.51), these were Italian high school students in Tuscany suggesting the one factor structure of the CNS. However, there is still no solid validation of the CNS on a representative sample, in fact, through this study, our intention is to validate CNS on a sample that is large both in number (*N* = 271) and age-range (34.70 years) and more generalized through- out Italy, with all of the 20 regions being represented. Therefore, our goal was to adapt and validate Mayer and Frantz’s Scale Mayer and Frantz (2004) in the Italian context as a contribution to climate change studies and pro-environmental behaviors. This research consisted of one main study focused on validating the factor structure of the questionnaire and aimed to explore the construct validity by assessing its convergent and discriminant validity. This validation opens new cultural perspectives as it can contribute to promote more studies about Italian attitudes and behaviors toward environment. In order to do this, we conducted a study to establish the internal consistency of the CNS scale and examine its convergent, discriminant, and predictive validity through empirical evidence.

## 2. Aims

In light of the growing interest in understanding the connection between humans and nature and the need for its evaluation, our study aimed to assess the psychometric validity and reliability of the Italian version of the CNS scale in a sample of Italian adults. Specifically, our objectives were as follows:

- Test the factorial validity using confirmatory factor analysis (CFA): We employed CFA to examine the factorial validity of the CNS scale, considering the model proposed by Mayer and Frantz (2004). We assessed whether the expected model fit the data using Hu and Bentler’s guidelines for various fit indices. We hypothesized that the Italian version of the CNS scale would exhibit the same factor structure as the original version.
- Assess the reliability and concurrent, convergent, and divergent validity: We examined the associations between the CNS scale and its correlates, including pro-environmental behavior (measured using the PEBS Questionnaire), moral disengagement with nature (measured using the Civilian Moral Disengagement Questionnaire), and individual levels of well-being (measured using the Mental Health Scale). Our hypothesis was that a higher connection to nature would be associated with greater consideration for the environment, increased environmental responsibility, and a positive relationship between well-being and affinity with nature.

## 3. Materials and methods

### 3.1. Participants

The sample for this study consisted of 271 Italian adults, with 44.3% females and 55% males. The mean age of the participants was 34.70 (SD = 13.584), ranging from 18 to 65 years. The sample size was determined following the guidelines proposed by Austin and Steyerberg (2015), as also observed in other studies conducted by Collister et al. (2023), Herrero-Montes et al. (2023), Huang et al. (2023), Stepovic et al. (2023), and Yamazaki et al. (2023). Table 1 presents the sociodemographic characteristics of the sample. All participants were recruited through the Prolific platform, which enabled the selection of a population based on specific sociodemographic criteria. The criteria ensured the inclusion of individuals of both genders, aged between 18 and 65 years. Efforts were made to include participants from various regional areas of Italy. Each participant received a reward of £2.25 for every 15 min of participation. Prior to the study, written informed consent was obtained from all participants, and a comprehensive explanation of the study procedure was provided. The experimental protocol of the study was approved by the local Ethics Committee of the Department of Psychology at the Catholic University of the Sacred Heart in Milan, Italy.

**Table 1:** Sociodemographic characteristics of the construction and validation samples.

Sociodemographic characteristics	Construction sample *N* = 271
Age, mean ± SD	34.70 ± 13.584
Gender	N (%)
Male	149 (55%)
Female	120 (44.3%)
Residence	N (%)
North Italy	170 (63.2%)
Centre Italy	35 (12.9%)
South Italy	32 (13.3%)
Sicily and Sardinia	29 (10.7%)
Outside Italy	–
Educational level	N (%)
Middle school or below	4 (1.5%)
High school	127(46.9%)
Graduate school	110(40.6%)
Postgraduate school Other	26 (9.6%) 4 (1.5%)
Employment status	N (%)
Student Workman	84 (31%) 3 (1.1%)
Employed Freelance	71 (26.2%) 36 (13.3%)
Unemployed Pensioner	30 (11.1%) 18 (6.6%)
Other	29 (10.7%)

### 3.2. Procedure and measures

The scale items underwent a process of back translation. Data were collected through an online survey administered on the Qualtrics platform from June 2022 to July 2022. Participants first provided sociodemographic information, including age, gender, residence, occupation, and level of study. Following that, they randomly completed four different questionnaires: the Connectedness to Nature Scale, the Mental Health Scale, the Civic Moral Disengagement Scale, and the Pro-Environmental Behaviors Scale.

#### 3.2.1. Connectedness to nature scale

The Connectedness to Nature Scale (CNS), developed by Frantz and Mayer (2014), consist of 14 items that are rated on a five-point Likert scale, ranging from “completely disagree” to “completely agree.” The CNS assesses the affective experience of individuals in relation to their connection with nature. This scale is widely used in the field of social and environmental psychology to measure the emotional connection that individuals feel toward the natural world. It aims to capture the extent to which individuals are emotionally and experientially connected to nature. The concept of connection with nature reflects the relationship between people and their environment. In the validation sample, the internal reliability of the CNS was found to be good, with a Cronbach’s alpha coefficient of 0.84.

#### 3.2.2. Pro-environmental Behaviors scale

The PEBS (Markle, 2013) measures people environmental attitudes, behaviors and values. It consists of 19 items. The scale will be used to assess convergent validity. The Pro-Environmental Behavior Scale encompasses various behaviors that can be categorized into four distinct types: the scale measures four factors: Conservation (CO; items 2–3–5-6), Environmental Citizenship (EC; items 8–9–10-11-12-13), Food (FO; items 14–15-16) and Transportation (TA; items 17–18-19). The behaviors incorporated in the scale are those recognized by environmental scientists as having significant consequences for the environment. Internal reliability in the validation sample was good (α = 0.71).

#### 3.2.3. Civilian moral disengagement

The DMC (Caprara et al., 2006) is a scale composed of 40 assertions with a five-step Likert rating that aims to assess the inclination to enact disengagement mechanisms in conjunction with various transgressions occurring in the course of everyday life, in different contexts, in various types of interpersonal relationships and with respect to various types of moral orders or codes. The DMC analyses the following aspects: Moral Justification (MJ; items 16–23–28-30-37), Euphemistic Labeling (EL; items 1–13–17-22-40), Advantageous Comparison (AC; items 5–15–26-29-35), Displacement of Responsibility (DR; items 2–6–20-25-34), Spread of Responsibility (SR; items 7–14–21-27-38), Distortion of Consequences (DC; 8–10–12-19-33), Attribution of Blame (AB; items4-11-18-24-31) and Dehumanization of the Victim (DV; items 3–9–32-36-39). The scale will be used to assess divergent validity. The internal reliability of the scale was found to be excellent (α = 0.91).

#### 3.2.4. Mental health scale

The Mental Health Scale (MHC), developed by Petrillo et al. (2015), assesses an individual’s levels of well-being by considering both hedonic and eudemonic dimensions. It is based on the assumption that individuals need to exhibit high levels of well-being in both dimensions to flourish in life, whereas low levels on both dimensions indicate a state of languishing. The questionnaire consists of 14 items rated on a six-point Likert scale. It measures emotional well-being (EWB; items 1–3), social well-being (SWB; items 4–8), and psychological well-being (PWB; items 9–14). The scale will be utilized to examine predictive validity. The internal reliability of the scale was good, with a Cronbach’s alpha coefficient of 0.9.

### 3.3. Data analysis

Confirmatory Factor Analysis (CFA) was employed to evaluate the factorial validity of the CNS, based on the model proposed by Greenberg et al. (2017). CFA analysis was used to examine the internal validity of the CNS and assess the psychometric properties of the scale. Goodness-of-fit indices were utilized, including the *χ*^2^/df ratio (≤ 3 for acceptable fit), Comparative Fit Index (CFI), Tucker Lewis Index (TLI), Root Mean Square Error of Approximation (RMSEA), and Standardized Root Mean Square Residual (SRMR). Following Hu and Bentler’s guidelines (Bentler, 1990; Hu and Bentler, 1999; Schermelleh-Engel et al., 2003), acceptable fit was indicated by CFI values ≥0.90, TLI values ≥0.90, RMSEA values between 0.05 and 0.08, and SRMR values ≤0.08. The CFA results demonstrated good fit across the various indices mentioned above. Furthermore, a multigroup CFA was conducted to examine whether the same number of factors was extracted across different groups.

### 3.4. Correlations

The validity of CNS was assessed by correlating (Pearson *r*) the CNS factors with theoretically related measures, namely PEBS to establish convergent validity. Second, we repeated the correlations between CNS and DMC to examine the divergent validity. Third we run the correlations between CNS and MHC to examine the predictive validity. Following Cohen’s guidelines (Cohen, 1988) we interpreted correlations as measures of the effect size.

## 4. Results

The total number of participants was 332 participants. Exclusion criteria were used to define the study sample: those who had not completed the entire administration of the questionnaires, those who did not answer or gave incomplete answers were excluded from the study. As a result, we collected a sample of 271subjects.

### 4.1. Reliability

The internal consistency of the CNS was good, as indicated by a Cronbach’s alpha coefficient of 0.89. Partial alpha coefficients further confirmed that the one-factor structure of the scale exhibited satisfactory internal consistency. No significant changes in overall reliability were observed, and no items were deemed necessary to be removed based on the analysis.

### 4.2. Confirmatory factor analysis

**Figure 1:**
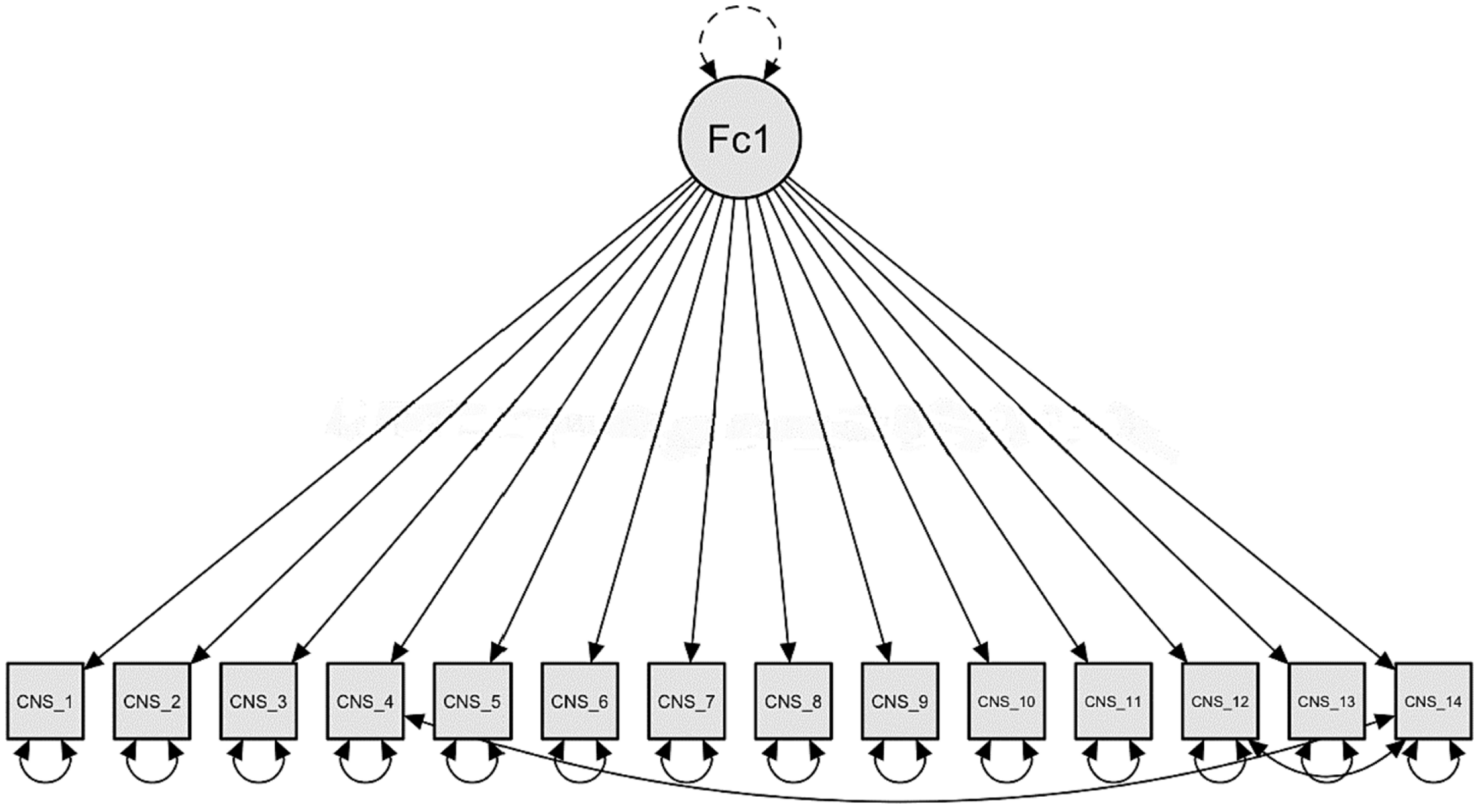
Model plot.

Confirmatory factor analysis (CFA) has been carried out and obtained results in all indices. Additionally, we checked for a correlation between elements using Bartlett’s test of sphericity (*χ*^2^ 158,718 df = 71, *p* < 0.001) and the Kaiser-Meyer-Olkin (KMO = 0.90). The CFA analysis revealed one factor structure was as the original scale proposed by Mayer and Frantz (2004). Further examination of the modification indices (MI) >10 suggested that the correlations between items 4 and 14 (MI = 20.409), 12 and 14 (MI = 16.241). Overall, the goodness-of-fit indices suggested that CFA analysis supported the internal consistency and validity of the factor model (Table 2) (Figure 1). Moreover, to investigate the effectiveness of the model across gender a multigroup CFA was carried out for women (N = 120) and men (N = 149). The CFA proved an adequate fit, suggesting factor invariance across gender (Table 3).

**Table 2:** Fit indices of connectedness to nature scale generated by CFA.

Index	Value
*X*^2^/df	2,235
Comparative fit index (CFI)	0.943
Tucker-Lewis index (TLI)	0.927
Root mean square error of approximation (RMSEA)	0.068
RMSEA 90% CI lower bound	0.054
RMSEA 90% CI upper bound	0.082
Standardized root mean square residual (SRMR)	0.044

**Table 3:** Goodness-of-fit indices generated by the multigroup CFA across gender.

Index	Value
*X*^2^/df	1,913
Comparative fit index (CFI)	0.913
Tucker-Lewis index (TLI)	0.89
Root mean square error of approximation (RMSEA)	0.083
Standardized root mean square residual (SRMR)	0.068

### 4.3. Correlations

#### 4.3.1. Convergent validity

The correlation between CNS and PEBS were significantly positive: *r* (CO) = 0.238 *p* < 0.001; *r* (EC) = 0.474, *p* < 0.001; r (FO) = 0.303, *p* < 0.001, *r* (FTA) = 0.205 *p* < 0.021. This demonstrate that CNS is a questionnaire assessing how an individual is aware of the nature.

#### 4.3.2. Divergent validity

The CNS inversely correlated with the DMC scale: *r* (MJ) = −0.094, *p* < 0.001; *r* (EL) = −0.20, *p* < 0.001; *r* (AC) = −0.249, *p* < 0.001; *r* (DR) = −0.161, *p* < 0.001; *r* (SR) = −0.296, *p* < 0.001; *r* (DC) = −0.145, *p* < 0.001; *r* (AB) = −0.228, *p* < 0.001; *r* (DV) = −0.304, *p* < 0.001. This showing that people connected to nature have a strong civic sense.

#### 4.3.3. Predictive validity

The CNS correlated with the MHC scale: *r* (EWB) = 0.332, *p* < 0.001; *r* (SWB) = 0.301, *p* < 0.001; *r* (PWB) = 0.410, *p* < 0.001. In fact, CNS is a questionnaire capable of measuring the relationship between connection to nature and psychophysical well-being.

## 5. Discussion

The aim of this study was to validate the Connectedness to Nature Scale (CNS) in the Italian context, which measures how which individuals feel connected to nature. To achieve this, a Confirmatory Factor Analysis (CFA) was conducted, providing insights into the factor structure of the scale in the Italian-speaking population. Additionally, this research opens up new avenues for exploring the relationship between humans and nature within the specific context of Italian-speaking regions, where there is a growing interest in this topic. To further strengthen the structure of the questionnaire it was not necessary to delete any items, but the original structure of the scale was maintained. The strength of the factor loadings and the goodness-of-fit indices indicated that the one factor structure was well-supported in this study. The model revealed a single factor concerning the connection to nature, this was consistent with the original version by Mayer and Frantz (2004). These findings demonstrate that the scale is well-constructed and maintains its intended purpose of assessing the emotional connection of individuals to the natural world, even after being translated into Italian. The scale aligns with the original conceptualization by Mayer and Frantz (2004), who described it as a measure of the affective aspect of the human-nature connection.

The CNS demonstrated a convergent validity consistent with the literature as evidenced by the correlations with the PEBS indices (Markle, 2013). The empirical evidence supports the idea that there is a link between people’s connection to nature and their pro-environmental behavior (PEB). Pro-environmental behavior refers to conscious actions taken to minimize one’s negative impact on the natural environment (Whitburn et al., 2020). A prevalent type of connection to nature involves the replenishment of adaptive resources that are crucial for an individual’s ongoing well-being, effectiveness, and environmental preservation (Hartig et al., 2014). In fact, the psychological advantages derived from experiencing nature may also contribute to concerns about the personal detrimental effects of environmental issues. These results are in accordance with our assumptions that connection to nature is strongly linked with the adoption of behaviors protecting the environment. A general perspective of this work, therefore, is that if people feel connected to nature, they will be less prone to damage it, motivate commitment to pro-environmental behaviors and encourage protection of the natural world (Tam, 2013; Frantz and Mayer, 2014; Olivos and Clayton, 2017; White et al., 2017). Conversely, disconnection to nature is associated to more general sense of apathy toward environmental degradation (Pyle, 2003).

Furthermore, as expected, negative correlations were found between CNS and DMC. People experiencing a high sense of civic moral disengagement are also feel a lower responsibility toward natural environment and less susceptible to understand the consequences of their actions (Kesebir and Kesebir, 2017). In psychology, moral disengagement, functioning as a cognitive process, allows individuals to engage in actions that are incongruent with their professed ethical principles and values. This capacity to separate their stated beliefs from their actions often giving rise to discrepancies between what one declares and how one behaves (Peeters et al., 2019). The mechanisms of moral disengagement enable humans to release themselves from inappropriate and reprehensible behavior. Every day we attend in implementing wrong actions toward the environment and we witness the effects of change on climate change to ecosystems and the consequent social and economic repercussions. An increasing number of people are worried about this situation, but it seems that this awareness alone is not enough to motivate individuals to adopt pro-environmental behaviors (Berenguer, 2007). Environmental moral disengagement represents a cognitive process wherein individuals distance themselves from ethical considerations when it comes to environmental actions. This disengagement allows them to justify actions that harm the environment, such as excessive consumption, pollution, or deforestation and poses a significant obstacle to effective environmental conservation. It is crucial to recognize that environmental moral disengagement is not an inherent trait but a cognitive mechanism that can be influenced and challenged (Ardoin et al., 2020; Ronen and Kerret, 2020).

Central to the issue is the notion that social responsibility, behavior, and sustainability are interconnected and can mutually shape and strengthen each other. Since moral disengagement plays an essential role in misconduct, it is important to understand how to enhance responsibility of individuals toward their behavior in order to promote environmental protective actions (Louv, 2008). Having a responsible attitude and behavior toward nature is a step toward preventive action toward nature, which is much more cost-effective than repairing the damage caused by over-exploitation and irresponsible interventions of human activities (Caciuc, 2014). People’s moral judgment often lacks the capability to recognize climate change as a significant moral concern due to its complex nature. Moreover, there is a reluctance of people to change their behavior and a temptation to escape responsibility (Peeters et al., 2019). The results suggest that moral disengagement can be mitigated by drawing from experiences in natural settings involving interpersonal interactions. This underscores the idea that collective interventions are essential in influencing individual behavior. Collective actions can encourage greater shared responsibility for upholding ethical norms. When individuals feel part of a group that promotes ethical behavior, they may be less inclined to rationalize moral disengagement as an isolated act (Panu, 2020; Whitburn et al., 2020; Seddon et al., 2021). Environmental education and proximity campaigns with nature could be used to try to solve this problem trying to motivate people to change their behavior and to be more responsible for their actions (Ardoin et al., 2020; Ronen and Kerret, 2020). The reduction in physical and emotional contact with nature can lead to decreased levels of care and interest in the planet, which in turn can have significant negative consequences on a global scale (Hartig et al., 2014).

A correlation was observed between CNS scores and all subdimensions of the MHC scale. These demonstrate that the connection with nature is closely linked to emotional, social and psychological well-being. Several studies have shown that connection and proximity to green spaces increases perceived well-being (Austin and Steyerberg, 2015), fosters cooperation within communities and promotes the development of pro-environmental attitudes. Human beings attribute to places a series of cognitive and emotional meanings that give rise to a specific quality of connection with the environment. This bond is characterized as a co-dependence between humans and the environment itself. Various researches (Ronen and Kerret, 2020; Huang et al., 2023) demonstrate that the physical characteristics of a place have a direct influence on the psychophysiological well-being or malaise of individuals and on the balance or imbalance between mind and body. Therefore, the physical environment is able to generate responses in individuals and to influence their perception of well-being or discomfort: the environment appears to be a determining factor in the way people feel and act. Fido and Richardson (2019) and Capaldi et al. (2014) have demonstrated that the connection with nature, mediated by empathy, is an innate affinity that stimulates people to behave correctly and respect the environment.

Related to this positive effect, Ronen and Kerret (2020) emphasized the important role of the environment and nature in fostering children and adolescents to improve their well-being in educational settings, suggesting that the quality of life is closely related to the quality of the environment from early ages.

A general perspective of this work concerns the idea that if people are more connected to nature, they will be less inclined to harm it. In general, there is evidence that there is a positive relationship between the CNS and actions in favor of the environment, which means that direct experiences with natural environments seem to have very profound emotional effects on people (Louv, 2008) and a greater commitment for nature could lead to a greater human interest in the protection of the environment. In the realm of psychological research, gaining insight into the motivational factors that drive individuals to develop environmental awareness and modify their behavior for its protection holds significant importance. By creating a sense of responsibility, promoting ethical norms, enhancing awareness, influencing policies, fostering a sense of belonging, supporting sustainable lifestyles, and driving positive behavioral change, collective actions represent a potent force in countering environmental moral disengagement. As we confront the pressing environmental challenges of our time, harnessing the collective power of individuals and communities is crucial to ensuring a sustainable future for our planet.

## 6. Conclusion

The CNS showed good psychometric properties as the original version by Mayer and Frantz (2004). Furthermore, the correlations supported that the CNS is a useful psychological tool for research dealing with emotional connection to nature, pro-environmental behaviors and individual civic engagement. Furthermore, these suggested that personal well-being is also related to a sense of connection to nature. We also investigated the validity and the reliability of the scale, demonstrating an adequate replication of one-factor structure of the original scale (Mayer and Frantz, 2004).

Mayer and Frantz (2004) described the CNS as a tool for gaging an individual’s emotional bond with nature. Assessing emotional connectedness to nature through this measure may serve as a significant predictor of pro-environmental attitudes, beliefs, and behaviors (Perrin and Benassi, 2009). In this light, future research may explore the relationship between the CNS and environmental education that, among various aims, deal with the development of pro-environmental practices for the well-being of the environment (Ardoin et al., 2020). Numerous studies have demonstrated that empathic engagement with the environment can facilitate the development of effective strategies aimed at promoting pro-environmental attitudes and behaviors (Berenguer, 2007; Tam, 2013; Fido and Richardson, 2019). A further future direction could investigate the relationship between connection to nature and empathy in order to understand whether these two psychological components may correlate each other. It may also be interesting to explore the association between CNS and the influence of some variables such as urban versus rural location or, socio-economic levels. Moreover, future studies could specifically address group invariance considering, besides gender as in this study, factors such as age and location. This would further inform about the scale sensitivity at an even more granular level.

## 7. Limitations

It is important to acknowledge some limitations of the study. One limitation pertains to the recruitment of participants, which relied on an online survey. While this method is commonly employed in research, it may inadvertently exclude individuals with limited internet access or technological difficulties. Moreover, the sample consists of subjects belonging more to Italian northern regions; despite the above limitation, the CNS appears well placed to measure levels of connection to nature. Furthermore, the questionnaire proves to be a reliable and psychometrically valid tool for research purposes, indicating the need for additional empirical investigations to further validate its efficacy.

## Data availability statement

The raw data supporting the conclusions of this article will be made available by the authors, without undue reservation.

## Ethics statement

The studies involving humans were approved by Commissione Etica per la Ricerca in Psicologia N:41–22. The studies were conducted in accordance with the local legislation and institutional requirements. The participants provided their written informed consent to participate in this study. Written informed consent was obtained from the individual (s) for the publication of any potentially identifiable images or data included in this article.

## Author contributions

CL and CD: substantial contributions to the conception or design of the work, or the acquisition, analysis, or interpretation of data for the work, and drafting the work or revising it critically for important intellectual content. FM, GG, and AM: substantial contributions to the conception or design of the work, or the acquisition, analysis, or interpretation of data for the work, drafting the work or revising it critically for important intellectual content, and provide approval for publication of the content. DM: provide approval for publication of the content. All authors contributed to the article and approved the submitted version.

## References

[ref1] Ardoin N. M. Bowers A. W. Gaillard E. (2020). Environmental education outcomes for conservation: a systematic review. Biol. Conserv. 241:108224. doi: 10.1016/j.biocon.2019.108224

[ref2] Arendt F. Matthes J. (2016). Nature documentaries, connectedness to nature, and pro-environmental behavior. Environ. Commun. 10, 453–472. doi: 10.1080/17524032.2014.993415

[ref3] Austin P. C. Steyerberg E. W. (2015). The number of subjects per variable required in linear regression analyses. J. Clin. Epidemiol. 68, 627–636. doi: 10.1016/j.jclinepi.2014.12.014, PMID: 25704724

[ref4] Barrera-Hernández L. F. Sotelo-Castillo M. A. Echeverría-Castro S. B. Tapia-Fonllem C. O. (2020). Connectedness to nature: its impact on sustainable behaviors and happiness in children. Front. Psychol. 11:276. doi: 10.3389/fpsyg.2020.00276, PMID: 32174866 PMC7054437

[ref5] Basile G. Cavallo A. (2020). Rural identity, authenticity, and sustainability in Italian inner areas. Sustainability 12:1272. doi: 10.3390/su12031272

[ref6] Beery T. H. Wolf-Watz D. (2014). Nature to place: rethinking the environmental connectedness perspective. J. Environ. Psychol. 40, 198–205. doi: 10.1016/j.jenvp.2014.06.006

[ref7] Bentler P. M. (1990). Comparative fit indexes in structural models. Psychol. Bull. 107, 238–246. doi: 10.1037/0033-2909.107.2.238 2320703

[ref8] Berenguer J. (2007). The effect of empathy in Proenvironmental attitudes and behaviors. Environ. Behav. 39, 269–283. doi: 10.1177/0013916506292937

[ref9] Berman M. G. Jonides J. Kaplan S. (2008). The cognitive benefits of interacting with nature. Psychol. Sci. 19, 1207–1212. doi: 10.1111/j.1467-9280.2008.02225.x 19121124

[ref10] Berto R. Barbiero G. Barbiero P. Senes G. (2018). An Individual’s connection to nature can affect perceived Restorativeness of natural environments some observations about Biophilia. Behav. Sci. 8:34. doi: 10.3390/bs8030034, PMID: 29510581 PMC5867487

[ref13] Caciuc V.-T. (2014). Ecocentric reflections on the realization of environmental education. Procedia. Soc. Behav. Sci. 137, 93–99. doi: 10.1016/j.sbspro.2014.05.258

[ref14] Capaldi C. A. Dopko R. L. Zelenski J. M. (2014). The relationship between nature connectedness and happiness: a meta-analysis. Front. Psychol. 5:e00976. doi: 10.3389/fpsyg.2014.00976, PMID: 25249992 PMC4157607

[ref15] Caprara G. V. Barbanelli C. Pastorelli C. Iafrate C. Beretta M. Steca P.. (2006). La misura del disimpegno morale nel contesto delle trasgressioni dell’agire quotidiano, 83–106.

[ref16] Cohen J. (1988). Statistical power analysis for the behavioral sciences (2nd) Erlbaum: Hillsdale.

[ref17] Collister D. Farrar M. Farrar L. Brown P. Booth M. Firth T.. (2023). Plasma exchange for ANCA-associated Vasculitis: an international survey of patient preferences. Kid. Med. 5:100595. doi: 10.1016/j.xkme.2022.100595, PMID: 36686273 PMC9851885

[ref18] Davis J. L. Green J. D. Reed A. (2009). Interdependence with the environment: commitment, interconnectedness, and environmental behavior. J. Environ. Psychol. 29, 173–180. doi: 10.1016/j.jenvp.2008.11.001

[ref19] Di Fabio A. Bucci O. (2016). Green positive guidance and Green positive life counseling for decent work and decent lives: some empirical results. Front. Psychol. 7:e00261. doi: 10.3389/fpsyg.2016.00261, PMID: 26973563 PMC4773606

[ref20] Di Fazio S. Modica G. (2018). Historic rural landscapes: sustainable planning strategies and action criteria. The Italian experience in the global and European context. Sustainability 10:3834. doi: 10.3390/su10113834

[ref21] Dutcher D. D. Finley J. C. Luloff A. E. Johnson J. B. (2007). Connectivity with nature as a measure of environmental values. Environ. Behav. 39, 474–493. doi: 10.1177/0013916506298794

[ref23] Fido D. Richardson M. (2019). Empathy mediates the relationship between nature connectedness and both callous and uncaring traits. Ecopsychology 11, 130–137. doi: 10.1089/eco.2018.0071

[ref24] Frantz C. M. Mayer F. S. (2014). The importance of connection to nature in assessing environmental education programs. Stud. Educ. Eval. 41, 85–89. doi: 10.1016/j.stueduc.2013.10.001

[ref25] Gilli G. Lovati C. Manzi F. Marchetti A. (2022). The one health approach: main psychological components. Ricerche Di Psicologia-Open Access.

[ref26] Gosling E. Williams K. J. H. (2010). Connectedness to nature, place attachment and conservation behaviour: testing connectedness theory among farmers. J. Environ. Psychol. 30, 298–304. doi: 10.1016/j.jenvp.2010.01.005

[ref27] Greenberg D. M. Kolasi J. Hegsted C. P. Berkowitz Y. Jurist E. L. (2017). Mentalized affectivity: a new model and assessment of emotion regulation. PLoS One 12:e0185264. doi: 10.1371/journal.pone.0185264, PMID: 29045403 PMC5646776

[ref28] Hartig T. Mitchell R. de Vries S. Frumkin H. (2014). Nature and health. Annu. Rev. Public Health 35, 207–228. doi: 10.1146/annurev-publhealth-032013-182443 24387090

[ref29] Herrero-Montes M. Fernández-de-las-Peñas C. Ferrer-Pargada D. Izquierdo-Cuervo S. Abascal-Bolado B. Valera-Calero J. A.. (2023). Association of Kinesiophobia with catastrophism and sensitization-associated symptoms in COVID-19 survivors with post-COVID pain. Diagnostics 13:847. doi: 10.3390/diagnostics13050847, PMID: 36899992 PMC10000376

[ref30] Hu L. Bentler P. M. (1999). Cutoff criteria for fit indexes in covariance structure analysis: conventional criteria versus new alternatives. Struct. Equ. Model. Multidiscip. J. 6, 1–55. doi: 10.1080/10705519909540118

[ref31] Huang W. Zhang F. Sun X. Yu Q. Huang J. Su Y.. (2023). Association between intimate partner psychological violence and psychological distress among nurses: the role of personality traits and social support. Front. Psychol. 13:1038428. doi: 10.3389/fpsyg.2022.1038428, PMID: 36710775 PMC9878691

[ref32] Kesebir S. Kesebir P. (2017). A growing disconnection from nature is evident in cultural products. Perspect. Psychol. Sci. 12, 258–269. doi: 10.1177/1745691616662473, PMID: 28346112

[ref33] Kesenheimer J. S. Greitemeyer T. (2021). Going Green (and not being just more pro-social): do attitude and personality specifically influence pro-environmental behavior? Sustainability 13:3560. doi: 10.3390/su13063560

[ref34] Louv R. (2008). Last child in the woods: Saving our children from nature-deficit disorder. London: Atlantic Books.

[ref35] Markle G. L. (2013). Pro-environmental behavior: does it matter how It’s measured? Development and validation of the pro-environmental behavior scale (PEBS). Hum. Ecol. 41, 905–914. doi: 10.1007/s10745-013-9614-8

[ref36] Mayer F. S. Frantz C. M. (2004). The connectedness to nature scale: a measure of individuals’ feeling in community with nature. J. Environ. Psychol. 24, 503–515. doi: 10.1016/j.jenvp.2004.10.001

[ref37] Menardo E. Brondino M. Pasini M. (2020). Adaptation and psychometric properties of the Italian version of the pro-environmental Behaviours scale (PEBS). Environ. Dev. Sustain. 22, 6907–6930. doi: 10.1007/s10668-019-00520-3

[ref38] Navarro O. Galharret J.-M. Olivos P. Loureiro A. Wittenberg I. Lemée C.. (2022). The brief version of the “connectedness to nature scale”: factorial structure and invariance study across seven European cities. Ecopsychology 14, 190–199. doi: 10.1089/eco.2021.0058

[ref39] Nilsson K. Bentsen P. Grahn P. Mygind L. (2019). De quelles preuves scientifiques disposons-nous concernant les effets des forêts et des arbres sur la santé et le bien-être humains? Sante Publique S1, 219–240. doi: 10.3917/spub.190.0219 31210482

[ref40] Nisbet E. K. Shaw D. W. Lachance D. G. (2020). Connectedness with nearby nature and well-being. Front. Sustain. Cities 2:18. doi: 10.3389/frsc.2020.00018

[ref41] Nisbet E. K. Zelenski J. M. (2013). The NR-6: a new brief measure of nature relatedness. Front. Psychol. 4:e00813. doi: 10.3389/fpsyg.2013.00813, PMID: 24198806 PMC3814587

[ref42] Olivos P. Clayton S. (2017). Self, nature and well-being: sense of connectedness and environmental identity for quality of life. Fleury-Bahi In G., Pol E. Navarro O. Handbook of environmental psychology and quality of life research Springer International Publishing: New York.

[ref43] Panu P. (2020). Anxiety and the ecological crisis: an analysis of eco-anxiety and climate anxiety. Sustainability 12:7836. doi: 10.3390/su12197836

[ref44] Peeters W. Diependaele L. Sterckx S. (2019). Moral disengagement and the motivational gap in climate change. Ethical Theory Moral Pract 22, 425–447. doi: 10.1007/s10677-019-09995-5

[ref45] Perrin J. L. Benassi V. A. (2009). The connectedness to nature scale: a measure of emotional connection to nature? J. Environ. Psychol. 29, 434–440. doi: 10.1016/j.jenvp.2009.03.003

[ref46] Petrillo G. Capone V. Caso D. Keyes C. L. M. (2015). The mental health continuum–short form (MHC–SF) as a measure of well-being in the Italian context. Soc. Indic. Res. 121, 291–312. doi: 10.1007/s11205-014-0629-3

[ref47] Picarelli L. Comegna L. Gariano Guzzetti Mercogliano Rianna. (2016). “Potential climate changes in Italy and consequences for land stability” in Slope safety preparedness for impact of climate change

[ref48] Pyle R. M. (2003). Nature matrix: reconnecting people and nature. Oryx 37, 206–214. doi: 10.1017/S0030605303000383

[ref49] Ray S. J. (2020). A field guide to climate anxiety: How to keep your cool on a warming planet. Berkeley: University of California Press.

[ref50] Ronen T. Kerret D. (2020). Promoting sustainable wellbeing: integrating positive psychology and environmental sustainability in education. Int. J. Environ. Res. Public Health 17:6968. doi: 10.3390/ijerph17196968, PMID: 32977640 PMC7579264

[ref51] Schermelleh-Engel K. Moosbrugger H. Müller H. (2003). Evaluating the fit of structural equation models: tests of significance and descriptive goodness-of-fit measures. Methods Psychol. Res. 8, 23–74.

[ref52] Seddon N. Smith A. Smith P. Key I. Chausson A. Girardin C.. (2021). Getting the message right on nature-based solutions to climate change. Glob. Chang. Biol. 27, 1518–1546. doi: 10.1111/gcb.15513, PMID: 33522071

[ref53] Stepovic M. Vekic S. Vojinovic R. Jovanovic K. Radovanovic S. Radevic S.. (2023). Analysis and forecast of indicators related to medical workers and medical Technology in Selected Countries of Eastern Europe and Balkan. Healthcare 11:655. doi: 10.3390/healthcare11050655, PMID: 36900660 PMC10000486

[ref54] Stern P. C. (2000). New environmental theories: toward a coherent theory of environmentally significant behavior. J. Soc. Issues 56, 407–424. doi: 10.1111/0022-4537.00175

[ref55] Tam K.-P. (2013). Dispositional empathy with nature. J. Environ. Psychol. 35, 92–104. doi: 10.1016/j.jenvp.2013.05.004

[ref56] Tam K.-P. (2022). Gratitude to nature: presenting a theory of its conceptualization, measurement, and effects on pro-environmental behavior. J. Environ. Psychol. 79:101754. doi: 10.1016/j.jenvp.2021.101754

[ref58] Whitburn J. Linklater W. Abrahamse W. (2020). Meta-analysis of human connection to nature and proenvironmental behavior. Conserv. Biol. 34, 180–193. doi: 10.1111/cobi.13381, PMID: 31251416 PMC7027494

[ref59] White M. P. Pahl S. Wheeler B. W. Depledge M. H. Fleming L. E. (2017). Natural environments and subjective wellbeing: different types of exposure are associated with different aspects of wellbeing. Health Place 45, 77–84. doi: 10.1016/j.healthplace.2017.03.008 28319857

[ref60] Yamazaki T. Saito Y. Yamashita D. Kitahara H. Kobayashi Y. (2023). Factors associated with impaired resistive reserve ratio and microvascular resistance reserve. Diagnostics 13, 1–13. doi: 10.3390/diagnostics13050950, PMID: 36900097 PMC10000988

[ref61] Zylstra M. J. Knight A. T. Esler K. J. Le Grange L. L. L. (2014). Connectedness as a Core conservation concern: an interdisciplinary review of theory and a call for practice. Spring. Sci. Rev. 2, 119–143. doi: 10.1007/s40362-014-0021-3

